# Machine learning-based colorectal cancer prediction using global dietary data

**DOI:** 10.1186/s12885-023-10587-x

**Published:** 2023-02-10

**Authors:** Hanif Abdul Rahman, Mohammad Ashraf Ottom, Ivo D. Dinov

**Affiliations:** 1 University of Michigan, Ann Arbor, USA; 2 PAPRSB Institute of Health Sciences, Universiti Brunei Darussalam, , Bandar Seri Begawan, Brunei; 3 Yarmouk University, Irbid, Jordan

**Keywords:** Colorectal cancer, Machine learning, Dietary information

## Abstract

**Background:**

Colorectal cancer (CRC) is the third most commonly diagnosed cancer worldwide. Active health screening for CRC yielded detection of an increasingly younger adults. However, current machine learning algorithms that are trained using older adults and smaller datasets, may not perform well in practice for large populations.

**Aim:**

To evaluate machine learning algorithms using large datasets accounting for both younger and older adults from multiple regions and diverse sociodemographics.

**Methods:**

A large dataset including 109,343 participants in a dietary-based colorectal cancer ase study from Canada, India, Italy, South Korea, Mexico, Sweden, and the United States was collected by the Center for Disease Control and Prevention. This global dietary database was augmented with other publicly accessible information from multiple sources. Nine supervised and unsupervised machine learning algorithms were evaluated on the aggregated dataset.

**Results:**

Both supervised and unsupervised models performed well in predicting CRC and non-CRC phenotypes. A prediction model based on an artificial neural network (ANN) was found to be the optimal algorithm with CRC misclassification of 1% and non-CRC misclassification of 3%.

**Conclusions:**

ANN models trained on large heterogeneous datasets may be applicable for both younger and older adults. Such models provide a solid foundation for building effective clinical decision support systems assisting healthcare providers in dietary-related, non-invasive screening that can be applied in large studies. Using optimal algorithms coupled with high compliance to cancer screening is expected to significantly improve early diagnoses and boost the success rate of timely and appropriate cancer interventions.

**Supplementary Information:**

The online version contains supplementary material available at 10.1186/s12885-023-10587-x.

## Introduction

In the current twenty-first century, the re-emergence of machine learning (ML) and advancement in artificial intelligence (AI) through data science provide unique opportunities to go beyond traditional statistical and research limitations, and advance health data analytics in solving healthcare challenges and ultimately improve the delivery of health services [1, 2].

One of the contemporary healthcare challenges is colorectal cancer (CRC). CRC is the third most commonly diagnosed malignancy after breast and lung cancers, and is also the second leading cause of cancer-related mortality worldwide [3, 4]. In 2020, an estimated 1.93 million new CRC cases were diagnosed, which accounts for 10% of the global cancer incidence [5]. The increasing number of global CRC cases could be attributed to successful population-based screening and surveillance programs that have been rapidly and actively implemented [6, 7]. Nonetheless, the number of CRC mortality is still high where 0.94 million deaths were recorded in 2020 that accounts for 9.4% of cancer deaths globally [5]. Active health screening and prevention of CRC activities have yielded an increasingly younger generation (below 50 years) of early-onset CRC in developed countries and overall increase in CRC incidence detection in developing and emerging economic nations [8, 9]. Increased pathophysiological understanding of CRC progression and the advancement of treatment options, including endoscopic and surgical interventions, radiotherapy, immunotherapy, and targeted chemotherapy, have effectively prolonged survival years and improved quality of life of CRC patients [9, 10]. The prognosis after CRC therapy is generally good when CRC is detected at a younger age, however, there is still huge public health challenges and financial burden associated with CRC [9]. In 2015, the economic cost of CRC in Europe due to hospital-care costs, loss of productivity, premature death, and costs of informal care was estimated at 19 billion euros [11]. Furthermore, the underlying mechanisms and risk factors of early-onset CRC pathological features are sporadic and not fully understood and require more research [9].

In this era of digital technology, the vast amount of high-quality CRC data (owing to an increase in the number of patients) can be rigorously collected through health information systems. This has enabled data science to offer a new avenue of enhancing knowledge of CRC through research and development. Currently, the extant evidence using machine-learning models have made great strides in predicting CRC based on available genetic-based data, which have shown that some CRC cases have a component of hereditary predisposition [12, 13]. However, genetic disorder is a permanent and non-modifiable risk factor. In contrast, dietary control is one of the most effective protective measures against CRC that the population can modify [4, 14] especially because CRC susceptibility is mainly resulting from adopting dietary lifestyle associated with globalization [15, 16]. With the globalization of the food industry and supply chain, it is thus important data science research to look into global diet features in relation to CRC prediction. In this study, we obtained global dietary-based data from publicly accessible databases and investigate the important dietary factors of predicting CRC labels using exploratory unsupervised and supervised ML-based models.

## Methods

### Dataset and data preprocessing

**Fig. 1:**
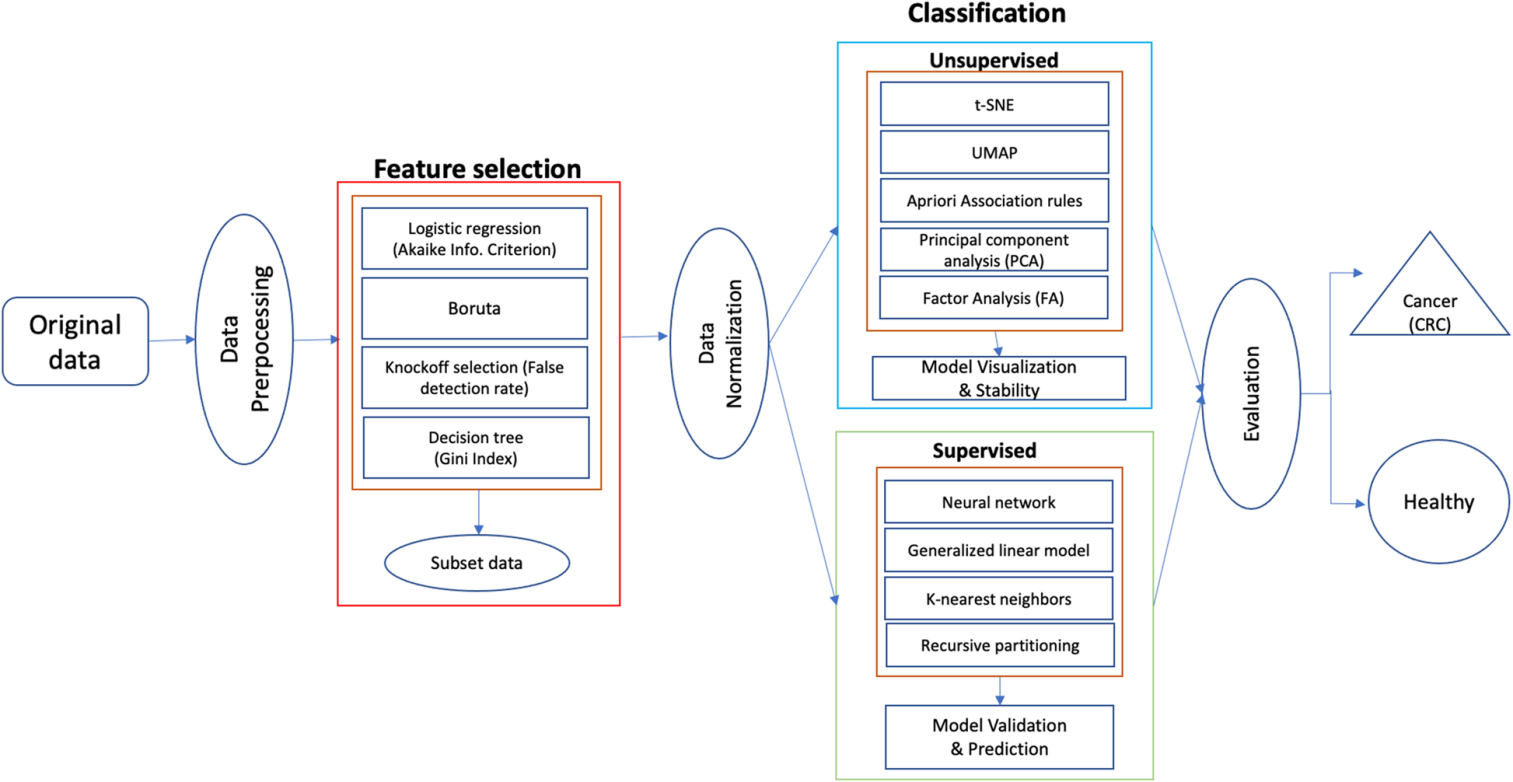
A schematic of the procedures undertaken in this study to classify CRC labels

Several end-to-end procedures were systematically performed, as illustrated in Fig. 1. Dietary-related colorectal cancer data was obtained from the Center for Disease Control and Prevention, Global Dietary database, and publicly accessible institutional sites [17, 18, 19, 20, 21, 22, 23]. The initial combined data contained 25 countries consisting of Argentina, Bangladesh, Bulgaria, Canada, China, Korea, Ecuador, Estonia, Ethiopia, Finland, Germany, India, Iran, Israel, Kenya, Malaysia, Mexico, Mozambique, Philippines, Portugal, Sweden, Tanzania, Italy, Japan, and the United States. The data collection methodology of these data sets were similar, i.e., cross-sectional and employed dietary questionnaires. The different sets of data were then merged and extrapolated based on the same dietary characteristics. Features that were not common across the data sets were excluded. This study only includes data sets that are of the English language. Features with different units of measurements were converted for standardization. A cleaning procedure was employed including removal of ineligible cases, duplicate characteristics, and features with more than 50% missing values (listwise deletion). At this stage, a total of 3,520,586 valid data remained. Due to computational limitations, a multi-stage, proportionate random sample of 109,342 were extracted for analysis, that maintains the percentage by country and CRC distribution, of which 7,326 (6.7%) cases were positive colorectal cancer labels that are derived for seven countries that comprised of Canada, India, Italy, South Korea, Mexico, Sweden, and United States. A sample size of 5,000 cases was sufficient to achieve a power of 0.8 [24]. Considering the computation ability of our machine could handle up to 110,000 data points, we randomly selected the maximum data load for this study. Table 1 presents the characteristics of the data.

**Table 1:** Data characteristics and sample statistics

	**Positive**		**Negative**		**Total**	
	n	%	n	%	n	%
**Overall**	7326	6.7	102,016	93.3	109,342	100
**Country**						
Canada	6014	5.5	103,328	94.5	31,381	28.7
India	4702	4.3	104,640	95.7	18,807	17.2
Italy	14,652	13.4	94,690	86.6	8966	8.2
South Korea	2406	2.2	106,936	97.8	16,292	14.9
Mexico	2406	2.2	106,936	97.8	10,387	9.5
Sweden	17,604	16.1	91,738	83.9	10,497	9.6
United States	11,153	10.2	98,189	89.8	12,902	11.8
**Gender**						
Male	7763	7.1	101,579	92.9	51,172	46.8
Female	6998	6.4	102,344	93.6	58,170	53.2
**Age (years)** [Mean (SD)]	48.9	(16.7)	36.4	(23.3)	41.6	(21.7)

**Fig. 2:**
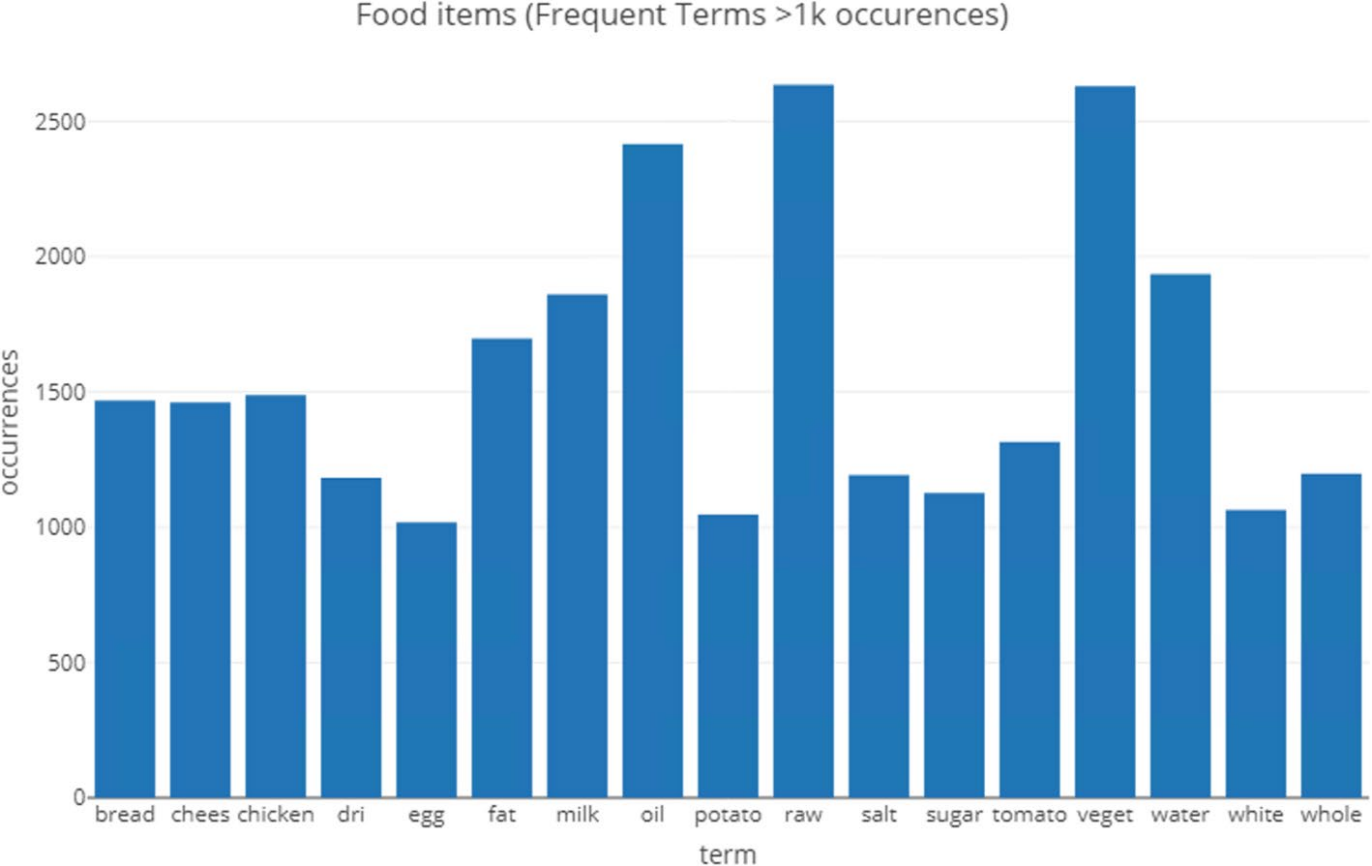
Frequent text items (1,000 occurrences) of the data

**Fig. 3:**
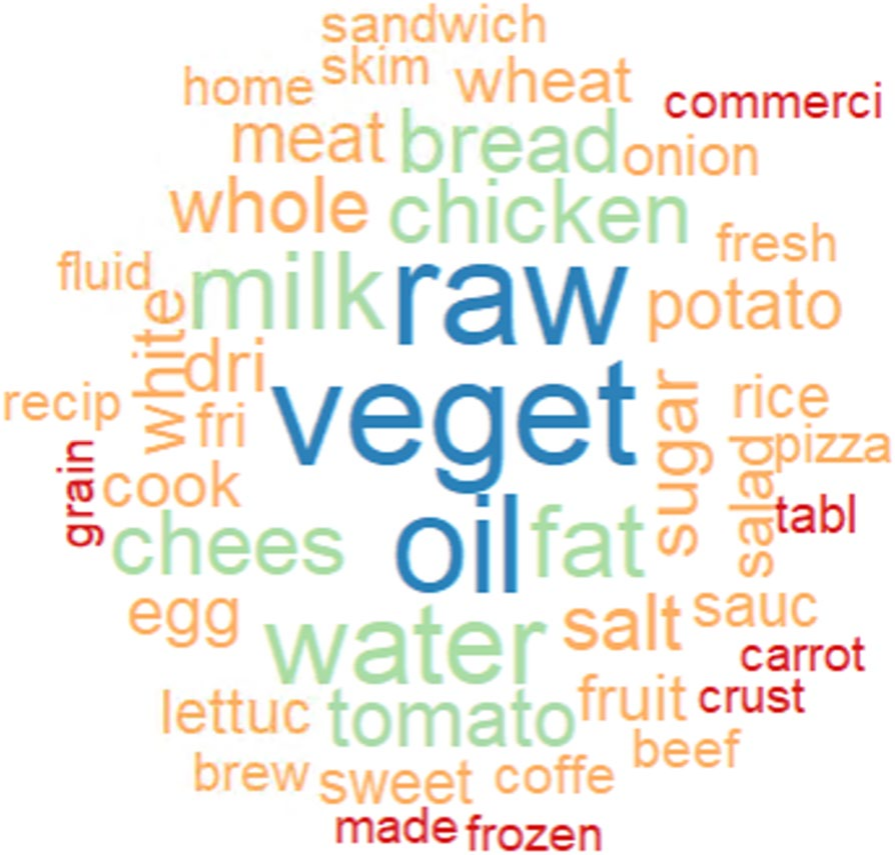
A word-cloud on the most frequent text items in the data set

Missing data in these valid cases was handled using multiple imputation techniques—MICE (Multivariate Imputation via Chained Equations) set at 10 multiple imputations to replace missing with predicted values, using R package *mice* [25]. The data set also consists of textual elements that describe the ingredients used such as milk, salt, chicken, and so on. Texts were converted into corpus objects and processed for standardization such as using English stop words, lower case, and removal of punctuation. The corpus item was then converted to a document term matrix to enable counting of most frequent terms occurring (Fig. 2), which are illustrated as a Wordcloud (Fig. 3). The important terms are converted into a data frame that is subsequently merged with the full data set. The dataset also has unbalanced binary CRC outcome, which was then re-balanced using the Synthetic Minority Oversampling Technique (SMOTE) [26].

### Feature selection

Two-step feature selection method was employed. Step one involves three separate procedures including Logistic regression (LR), Boruta, and Knockoff selection. LR was used to screen each single index out to reduce redundant features by computing a stepwise iterative process of forward addition (adding important features to a null set of features) and backward elimination (removing worst-performing features from the list of complete features) using the stepAIC function in the MASS package [27]. Variable selection was determined by the most significant features (*p* < 0.05) in the most parsimonious model with the lowest Akaike Information Criterion (AIC). Next, a randomized wrapper method, Boruta, which iteratively removes features that are statistically not significant and relevant than that of random probes, was employed [28]. Finally, the Knockoff selection based on the Benjamini–Hochberg False Discovery Rate method was implemented, that controls for expected proportion of false rejection of features in multiple significance testing [29], which could be expressed as follows:

$$\underset{\text{False Discovery Rate}}{\underbrace{FDR}} = \underset{\text{expectation}}{\underbrace{E}}\ \ \underset{\text{False DIscovery Proportion}}{\underbrace{\left(\frac{\#\text{FalsePositive}}{\text{total number of selected Features}}\right)}}$$

which determines the final selection based on variable importance using the Gini Index that is expressed as follows:

$$GI=\sum_{k}{p}_{k}\left(1-{p}_{k}\right)=1-\sum_{k}{p}_{k}^{2},$$

where $k$ is the number of classes.

The data set with the finalized features then was further processed using data normalization to avoid effects of extreme numeric ranges and to help obtain higher classification accuracy [30, 31]. The features were scaled as follows:

$${V}^{\mathrm{^{\prime}}}\hspace{0.17em}=\frac{V-\hspace{0.25em}Min\hspace{0.17em}}{Max-\hspace{0.17em}Min},$$

where ${V}^{\mathrm{^{\prime}}}$ is the scale value corresponding to the original value $\mathrm{V}$, and $Min$ and $Max$ are the upper and lower range limits.

Finally, the features that intersect among the two-step variable selection procedures were selected as the most salient features to be used for unsupervised and supervised classifications.

### Unsupervised techniques

Four types of unsupervised machine learning for non-linear relationship were used to explore the dimensions of the data including t-distributed stochastic Neighbor embedding (t-SNE), uniform manifold approximation and projection (UMAP), Apriori association rules, principal component analysis (PCA), and factor analysis (FA) [31, 32].

t-SNE technique is a machine learning strategy for nonlinear dimensionality reduction that is useful for embedding high-dimensional data into lower-dimensional spaces. If the high dimensional data ($N$ D) is ${x}_{1},{x}_{2},...,{x}_{N}$ then, for each pair (${x}_{i},{x}_{j}$), t-SNE estimates the probabilities ${p}_{i,j}$ that are proportional to their corresponding similarities, ${p}_{j|i}$:

$${p}_{j|i}=\frac{{\text{exp}}\left(\frac{-||{x}_{i}-{x}_{j}|{|}^{2}}{2{\sigma }_{i}^{2}}\right)}{\sum_{k\ne i}{\text{exp}}\left(\frac{-||{x}_{i}-{x}_{k}|{|}^{2}}{2{\sigma }_{i}^{2}}\right)} .$$

t-SNE performs a binary search for the value ${\sigma }_{i}$ that produces a predefined value $perp$. The perplexity ($perp$) of a discrete probability distribution, $p$, is defined as an exponential function of the entropy, $H(p)$, over all discrete events: $perp(x)={2}^{H(p)}={2}^{-\sum_{x}p(x){\text{log}}_{2}p(x)}$.

UMAP relies on local approximations of patches on the manifold to construct local fuzzy simplicial complex (topological) representations of the high dimensional data. For example, if $S1$ the set of all possible 1-simplexes, let’s denote by $\omega (e)$ and $\omega \mathrm{^{\prime}}(e)$ the weight functions of the 1-simplex $e$ in the high dimensional space and the corresponding lower dimensional counterpart. Then, the cross-entropy measure for the 1-simplexes is:

$$\sum_{e\in E}\left[\underset{\text{attractive force}}{\underbrace{{\omega \left(e\right)\mathrm{log}\left(\frac{\omega (e)}{\omega^{\prime}(e)}\right)}}}+\underset{\text{repulsive force}}{\underbrace{{\left(1-\omega \left(e\right)\right)\mathrm{log}\left(\frac{1-\omega (e)}{1-\omega^{\prime}(e)}\right)}}}\right].$$

The iterative optimization process would minimize the objective function composed of all cross entropies for all simplicial complexes using a strategy like stochastic gradient descent.

The optimization process balances the push–pull between the *attractive forces* between the points favoring larger values of $\omega \mathrm{^{\prime}}(e)$ (that correspond to small distances between the points), and the *repulsive forces* between the ends of $e$ when $\omega (e)$ is small (that correspond to small values of $\omega \mathrm{^{\prime}}(e)$.

The Apriori algorithm is based on a simple apriori belief that *all subsets of a frequent item-set must also be frequent*. We can measure a rule’s importance by computing its support and confidence metrics. The support and confidence represent two criteria useful in deciding whether a pattern is “valuable.” By setting thresholds for these two criteria, we can easily limit the number of interesting rules or item-sets reported.

For item-sets $X$ and $Y$, the support of an item-set measures how (relatively) frequently it appears in the data:

$$support(X)=\frac{count(X)}{N},$$

where *N* is the total number of transactions in the database and *count(X)* is the number of observations (transactions) containing the item-set *X*.

In a set-theoretic sense, the union of item-sets is an item-set itself. In other words, if $Z=X,Y=X\cup Y$, then

$$support(Z)=support(X,Y).$$

For a given rule $X\to Y$, the rule's confidence measures the relative accuracy of the rule:

$$confidence(X\to Y)=\frac{support(X,Y)}{support(X)}.$$

The confidence measures the joint occurrence of *X* and *Y* over the *X* domain. If whenever *X* appears *Y* tends to also be present, then we will have a high $confidence(X\to Y)$.

Note that the ranges of the support and the confidence are $0\le support,\hspace{0.25em}confidence\le 1$.

PCA (principal component analysis) is a mathematical procedure that transforms a number of possibly correlated variables into a smaller number of uncorrelated variables through a process known as orthogonal transformation. In general, the formula for the first PC is $p{c}_{1}={a}_{1}^{T}X=\sum_{i=1}^{N}{a}_{i,1}{X}_{i}$ where ${X}_{i}$ is a $n\times 1$ vector representing a column of the matrix $X$ (representing a total of n observations and N features). The weights ${a}_{1}=\{{a}_{1,1},{a}_{2,1},...,{a}_{N,1}\}$ are chosen to maximize the variance of $p{c}_{1}$. According to this rule, the ${k}^{th}$ PC is $p{c}_{k}={a}_{k}^{T}X=\sum_{i=1}^{N}{a}_{i,k}{X}_{i}$. ${a}_{k}=\{{a}_{1,k},{a}_{2,k},...,{a}_{N,k}\}$ has to be constrained by more conditions:

- Variance of $p{c}_{k}$ is maximized
- $$Cov(p{c}_{k},p{c}_{l})=0$$, $$\forall 1\le l<k$$
- ${a}_{k}^{T}{a}_{k}=1$ (the weights vectors are unitary)

FA optimization relies on iterative perturbations with full-dimensional Gaussian noise and maximum-likelihood estimation where every observation in the data represents a sample point in a higher dimensional space. Whereas PCA assumes the noise is spherical, Factor Analysis allows the noise to have an arbitrary diagonal covariance matrix and estimates the subspace as well as the noise covariance matrix.

Under FA, the centered data can be expressed in the following from:

$${x}_{i}-{\mu }_{i}={l}_{i,1}{F}_{1}+...+{l}_{i,k}{F}_{k}+{\epsilon }_{i}=LF+{\epsilon }_{i},$$

where $i\in 1,...,p$, $j\in 1,...,k$, $k<p$ and ${\epsilon }_{i}$ are independently distributed error terms with zero mean and finite variance.

### Supervised classifiers

The data was split into 80% for training and 20% for testing. The data was trained using machine learning (ML) algorithms including neural network (Neuralnet), k-nearest neighbors (kNN), generalized linear model (GLM), and recursive partitioning (Rpart).

Neuralnet model mimics the biological brain response to multisource stimuli (inputs). When we have three signals (or inputs) ${x}_{1}$, ${x}_{2}$ and ${x}_{3}$, the first step is weighting the features ($w$’s) according to their importance. Then, the weighted signals are summed by the “neuron cell” and this sum is passed on according to an activation function denoted by **f**. The last step is generating an output **y** at the end of the process. A typical output will have the following mathematical relationship to the inputs.

$$y(x)=f\left(\sum_{i=1}^{n}{w}_{i}{x}_{i}\right).$$

kNN classifier performs two steps calculations. For a given $k$, a specific similarity metric $d$, and a new testing case $x$,

- Runs through the whole training dataset ($y$) computing $d(x,y)$. Let $A$ represent the $k$ closest points to $x$ in the training data $y$.
- Estimates the conditional probability for each class, which corresponds to the fraction of points in $A$ with that given class label. If $I(z)$ is an indicator function

$$I(z)=\left\{\begin{array}{ll}1& z=true\\ 0& otherwise\end{array}\right.$$, then the testing data input $x$ gets assigned to the class with the largest probability, $P(y=j|X=x)$:

$$P(y=j|X=x)=\frac{1}{k}\sum_{i\in A}I({y}^{(i)}=j).$$

Generalized linear model, specifically, logistic regression, is a linear probabilistic classifier. It takes in the probability values for binary classification, in this case, positive (0) and negative (0) mental well-being and estimates class probabilities directly using the logit transform function [33].

Recursive partitioning (Rpart) is a decision tree classification technique that works well with variables with definite ordering and unequal distances. The tree is built similarly as a random forest with a resultant complex model, however, Rpart procedure also consists of a cross-validation stage to trim back the full tree into nested terminals. The final model of the sub-tree provides the decision with the ‘best’ or lowest estimated error [34].

### Model validation and performance assessment

Unsupervised techniques were evaluated based on the model visualization, as the best way to determine suitability of the models. Whereas, the ML-classifiers used specific parameters. The caret package was used for automated parameter tuning with *repeatedcv* method set at 15-folded cross-validation re-sampling that was repeated with 10 iterations [35]. The *k*-fold validation results and values were then used to calculate the confusion matrix that determines the measures of sensitivity, specificity, kappa, and accuracy. These measures were used to evaluate the performance of the ML-model classifiers. These measures were calculated as follows:

$$sensitivity=\frac{TP}{TP+FN}.$$

$$specificity=\frac{TN}{TN+FP}.$$

$$kappa=\frac{P(a)-P(e)}{1-P(e)}.$$

$$accuracy=\frac{TP+TN}{TP+TN+FP+FN}=\frac{TP+TN}{\text{Total number of observations}}$$

where, True Positive(TP) is the number of observations that are correctly classified as “yes” or “success.” True Negative(TN) is the number of observations that are correctly classified as “no” or “failure.” False Positive(FP) is the number of observations that are incorrectly classified as “yes” or “success.” False Negative(FN) is the number of observations that are incorrectly classified as “no” or “failure” (Dinov 2018).

## Results

### Feature importance

**Fig. 4:**
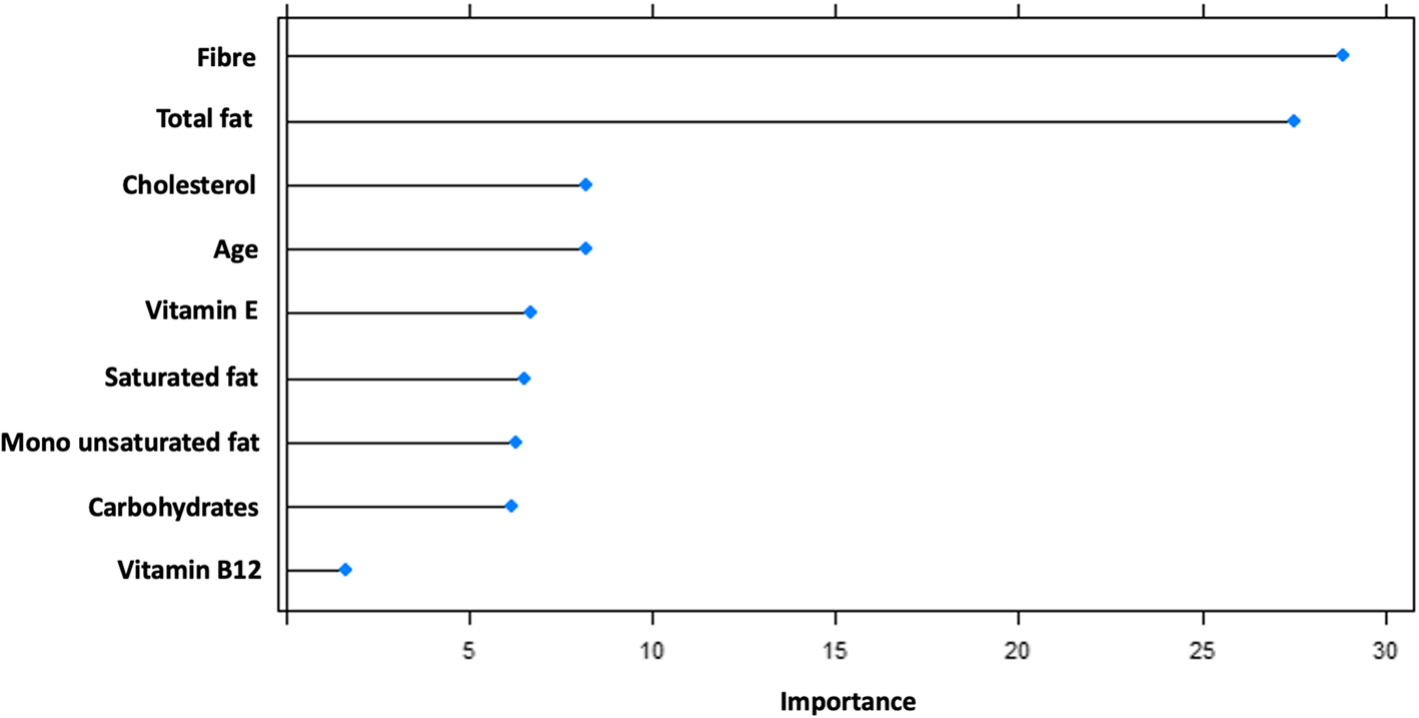
Variable importance plot showing contribution of features to predicting colorectal cancer

The common features derived from the procedures of variable selection yielded ten salient variables (Fig. 4) that are important contributors of CRC including, by order of importance, fiber, total fat, cholesterol, age, vitamin E, saturated fats, monounsaturated fats, carbohydrates, and vitamin B12. These features were used in the next step of machine learning modeling.

### Unsupervised learning

**Fig. 5:**
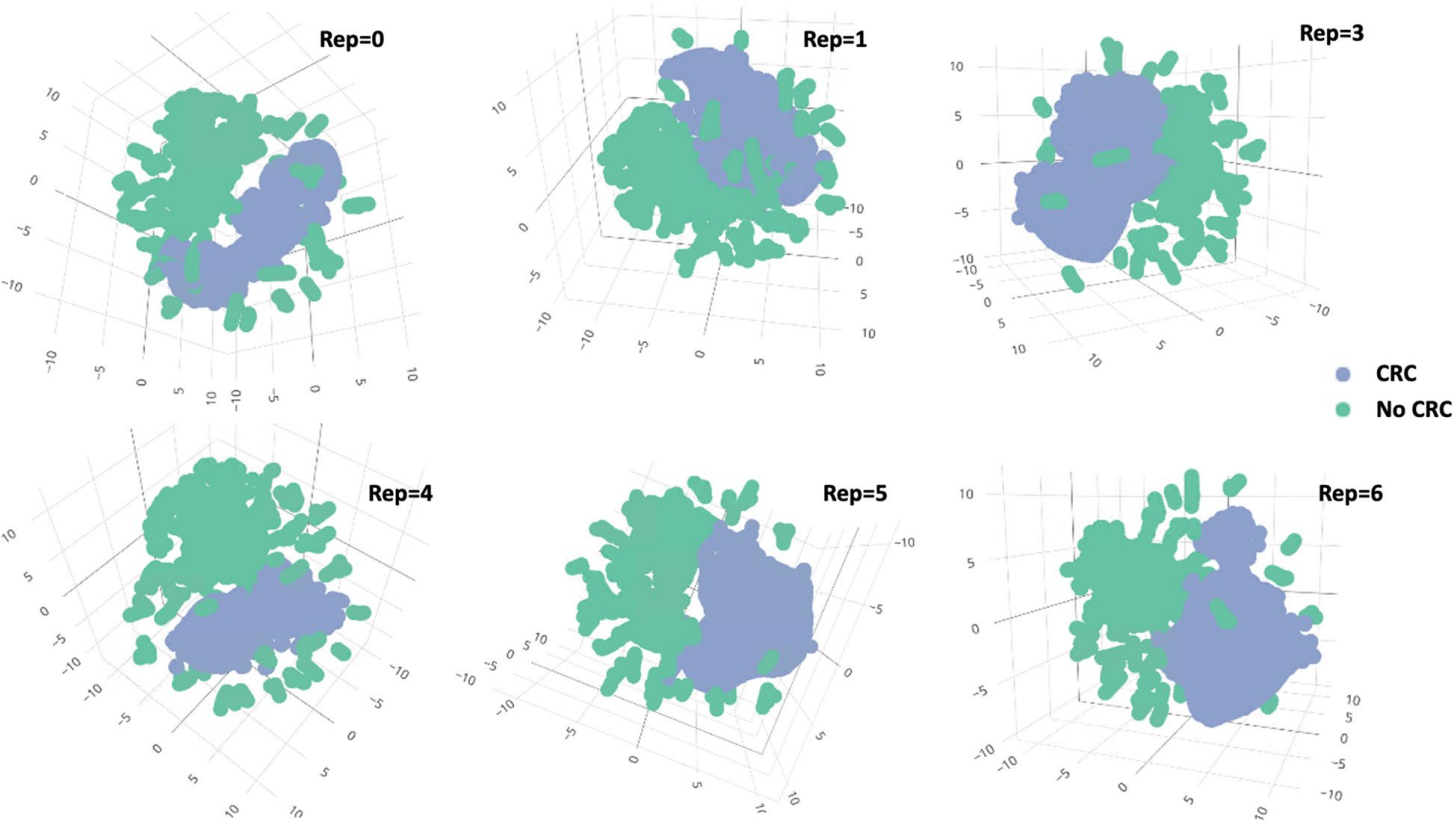
Stability of t-SNE 3D embedding (Perplexity = 50) with six repeated (Rep) computations of the classification of no- and yes- colorectal cancer (CRC) labels

**Fig. 6:**
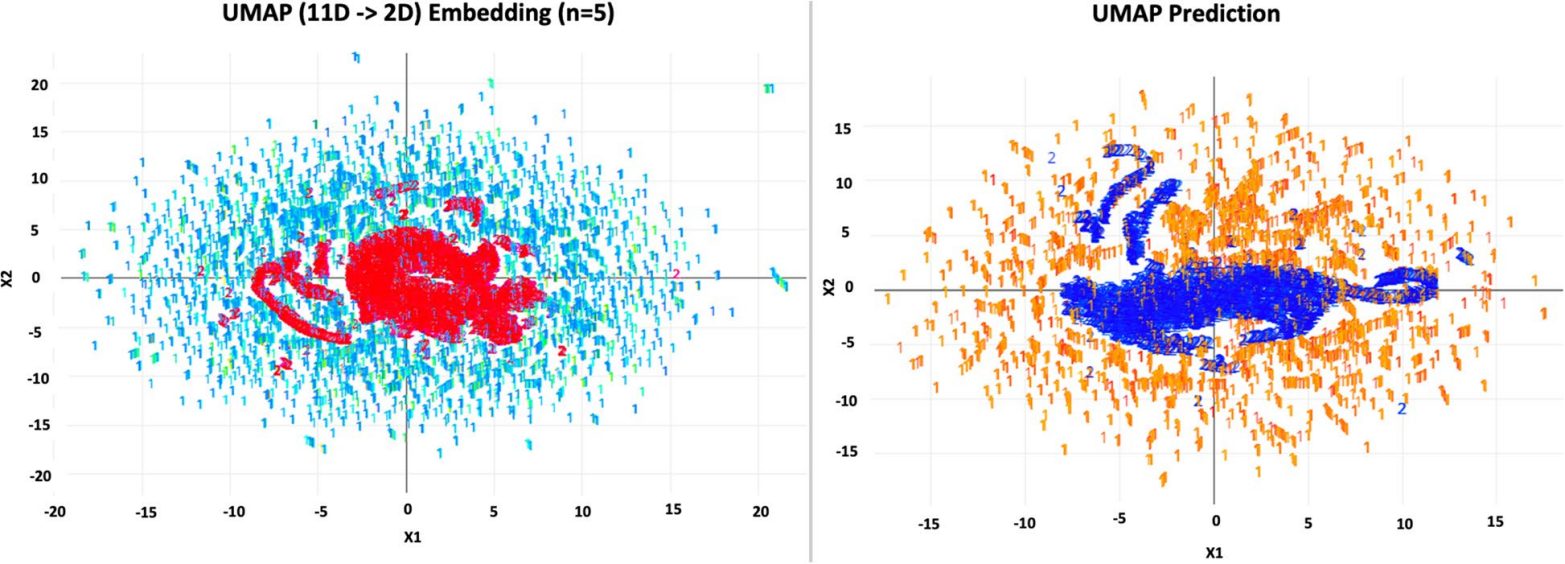
Uniform Manifold Approximation (UMAP) 2D embedding model (n-neighbor = 5) (L) and UMAP prediction on testing data (R) in the classification of no- and yes- colorectal cancer labels

**Fig. 7:**
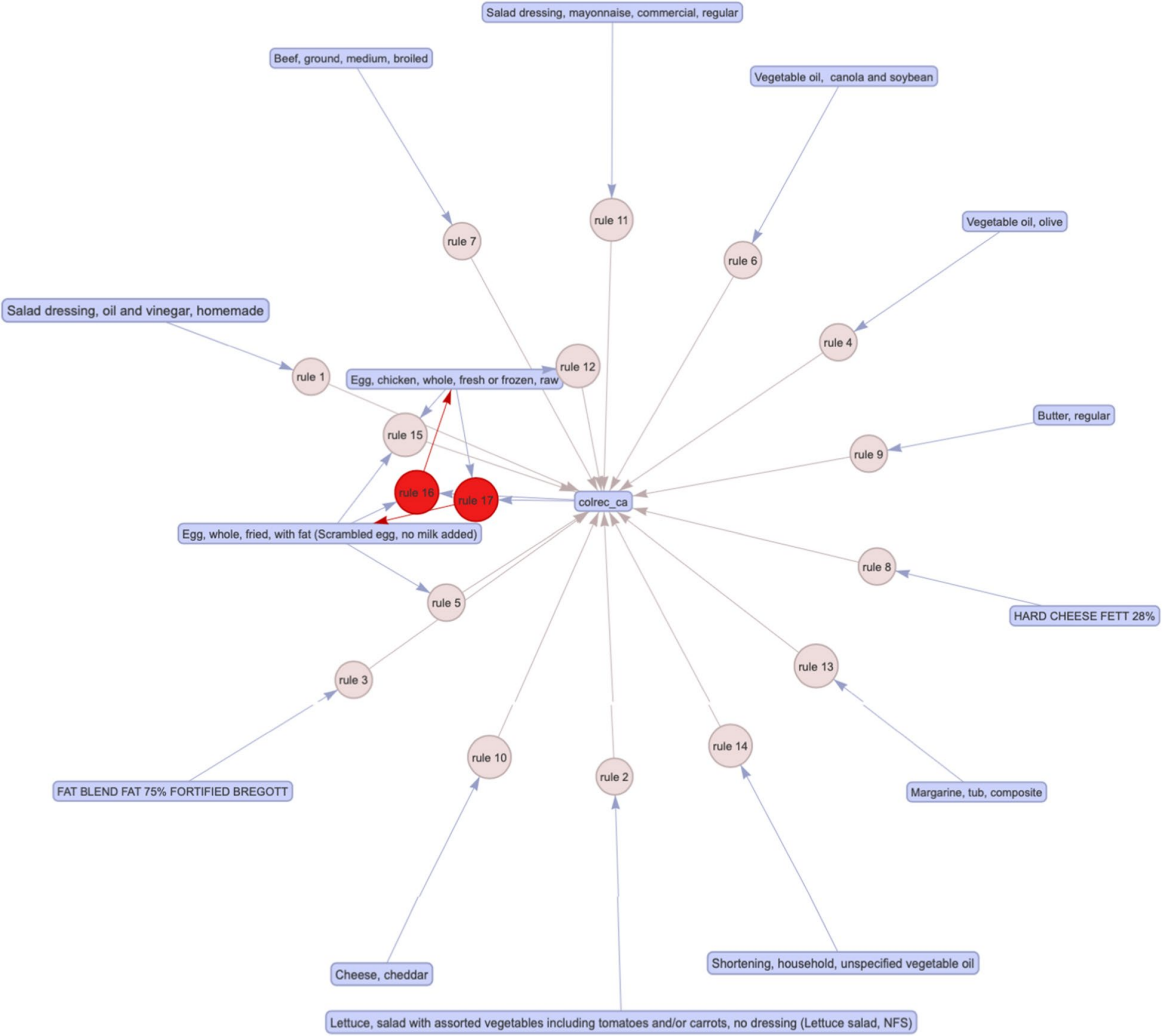
Apriori association rules of text features that are associated with the labelled yes colorectal cancer (“colrec_ca”) (See Supplementary 1 for this interactive html widget)

**Fig. 8:**
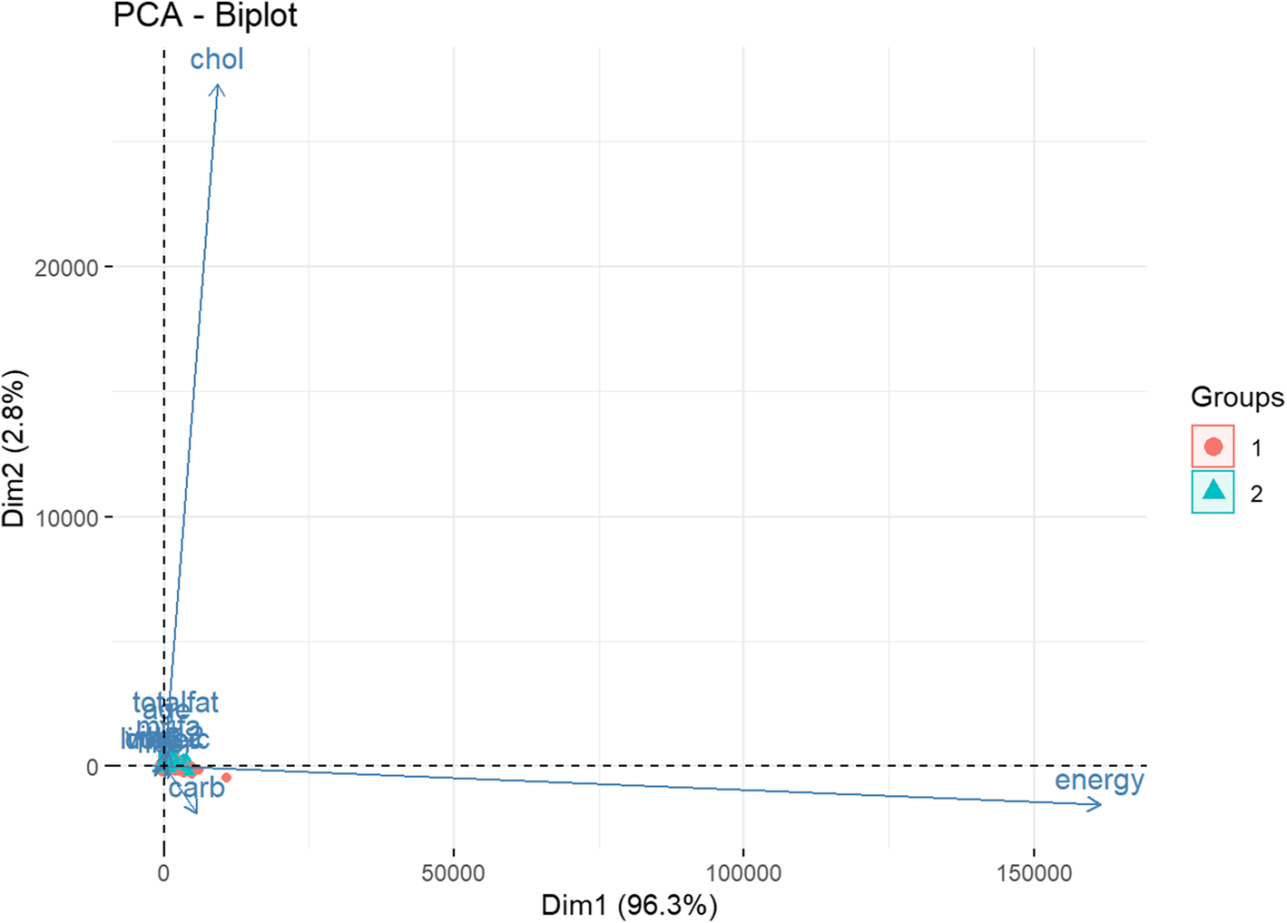
A bi-plot of Principal component analysis on the most optimal number of dimensions in the data where Group 1 is no cancer label and Group 2 is the cancer label

Among the unsupervised classifiers, t-SNE (Fig. 5) was the best performer. By visual inspection, t-SNE has maintained good stability of classifying positive CRC labels over several repeated computations. UMAP (Fig. 6) prediction also appears to be able to distinguish positive and negative CRC labels. Apriori association rules (Fig. 7) were able to map the textual features correlated to positive CRC labels, and the text items, by order of count, are listed in Table 2. PCA (Fig. 8) and FA (Table 3) showed that the data could be reduced to two dimensions where CRC is negatively correlated with fiber and carbohydrates, and positively correlated with the rest of the features.

**Table 2:** Summary description of apriori association rules of no colorectal cancer (“No_colrec_ca”) label

lhs	rhs	Support	Count
{Shortening, household, unspecified vegetable oil}	{colrec_ca}	0.062	3870
{Margarine, tub, composite}	{colrec_ca}	0.045	2818
{Egg, chicken, whole, fresh or frozen, raw}	{colrec_ca}	0.036	2277
{Cheese, cheddar}	{colrec_ca}	0.030	1903
{Salad dressing, mayonnaise, commercial, regular}	{colrec_ca}	0.029	1821
{HARD CHEESE FETT 28%}	{colrec_ca}	0.029	1804
{Butter, regular}	{colrec_ca}	0.028	1777
{FAT BLEND FAT 75% FORTIFIED BREGOTT}	{colrec_ca}	0.022	1360
{Beef, ground, medium, broiled}	{colrec_ca}	0.018	1150
{Vegetable oil, canola and soybean}	{colrec_ca}	0.018	1126
{Egg, whole, fried, with fat (Scrambled egg, no milk added)}	{colrec_ca}	0.016	998
{Vegetable oil, olive}	{colrec_ca}	0.015	971
{Lettuce, salad with assorted vegetables including tomatoes and/or carrots, no dressing (Lettuce salad, NFS)}	{colrec_ca}	0.014	905
{Egg, whole, fried, with fat (Scrambled egg, no milk added)} = > {Egg, chicken, whole, fresh or frozen, raw}	{colrec_ca}	0.012	751
{Egg, chicken, whole, fresh or frozen, raw} = > {Egg, whole, fried, with fat (Scrambled egg, no milk added)}	{colrec_ca}	0.012	751
{colrec_ca,Egg, whole, fried, with fat (Scrambled egg, no milk added)} = > {Egg, chicken, whole, fresh or frozen, raw}	{colrec_ca}	0.012	751
{colrec_ca,Egg, chicken, whole, fresh or frozen, raw} = > {Egg, whole, fried, with fat (Scrambled egg, no milk added)}	{colrec_ca}	0.012	751
{Egg, chicken, whole, fresh or frozen, raw, Egg, whole, fried, with fat (Scrambled egg, no milk added)}	{colrec_ca}	0.012	751
{Salad dressing, oil and vinegar, homemade}	{colrec_ca}	0.011	674

**Table 3:** Two-factor model in the dimensionality reduction procedure of the colorectal cancer data

Factor Analysis	Two-factor model	
	**Factor1**	**Factor2**
Age	0.178	
Energy	0.433	0.525
carbohydrates	-0.121	0.972
fiber	-0.118	0.703
Total fat	0.990	0.123
Mono unsaturated fats	0.946	0.103
Omega-6	0.512	
cholesterol	0.483	
Vitamin B12	0.164	0.204
Linoleic acid	0.566	
Colorectal cancer	0.655	

### Supervised learning

#### Model evaluation

**Fig. 9:**
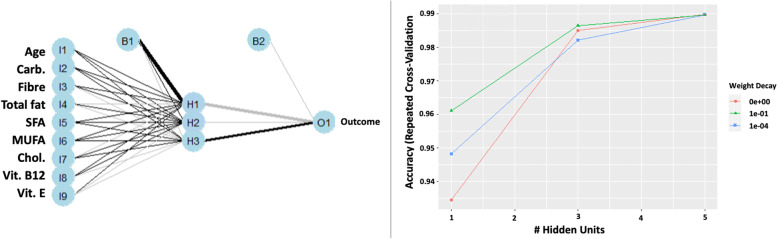
A schematic of a neural network with a single hidden layer with three hidden nodes (L) and weight decay of optimal hidden node parameter using repeated cross-validation (R)

**Fig. 10:**
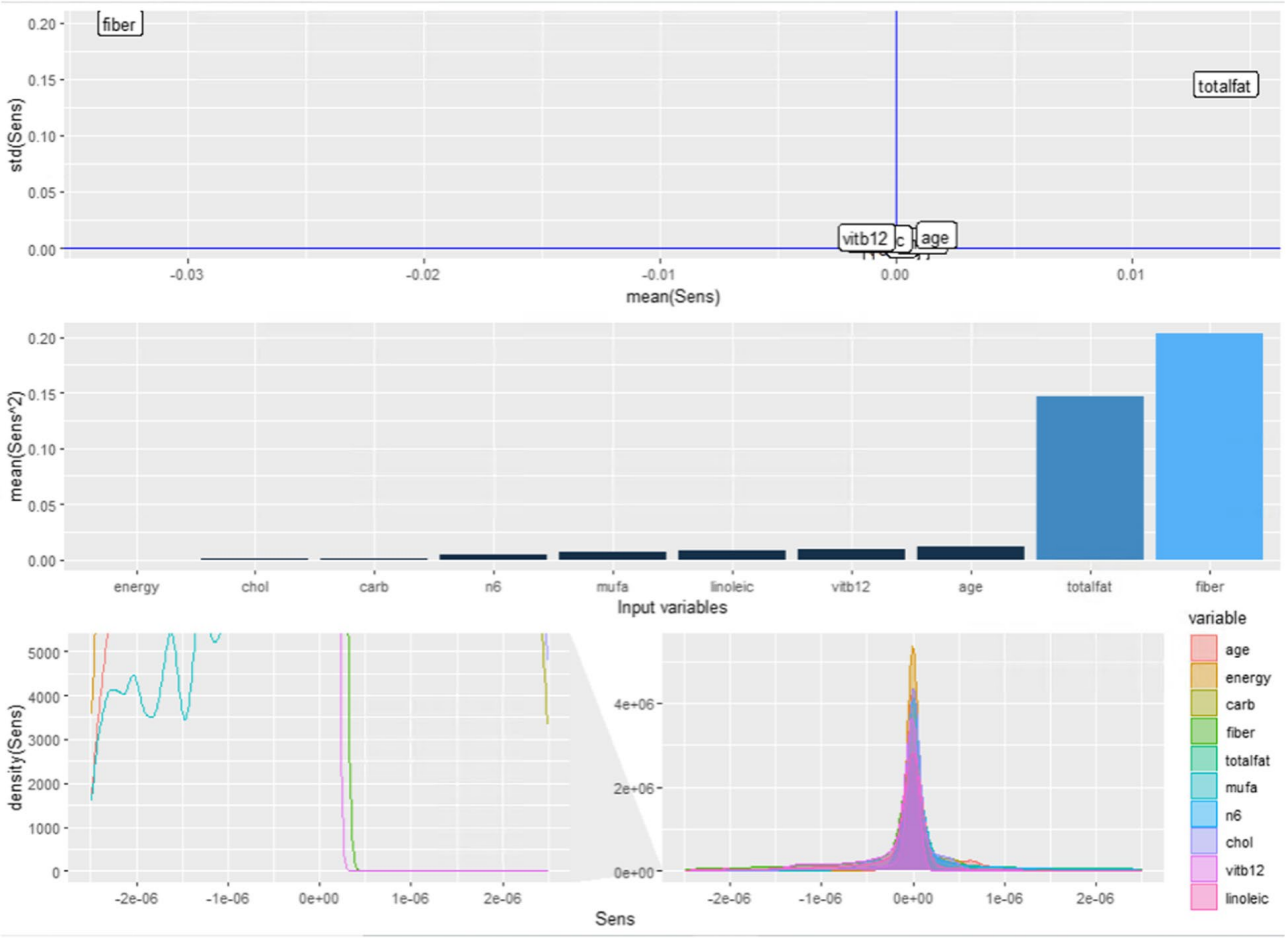
Sensitivity analysis of the three hidden node neural network model in relation to the mean and standard deviation (top), mean square difference among the input variables (middle), and density plots (bottom)

**Fig. 11:**
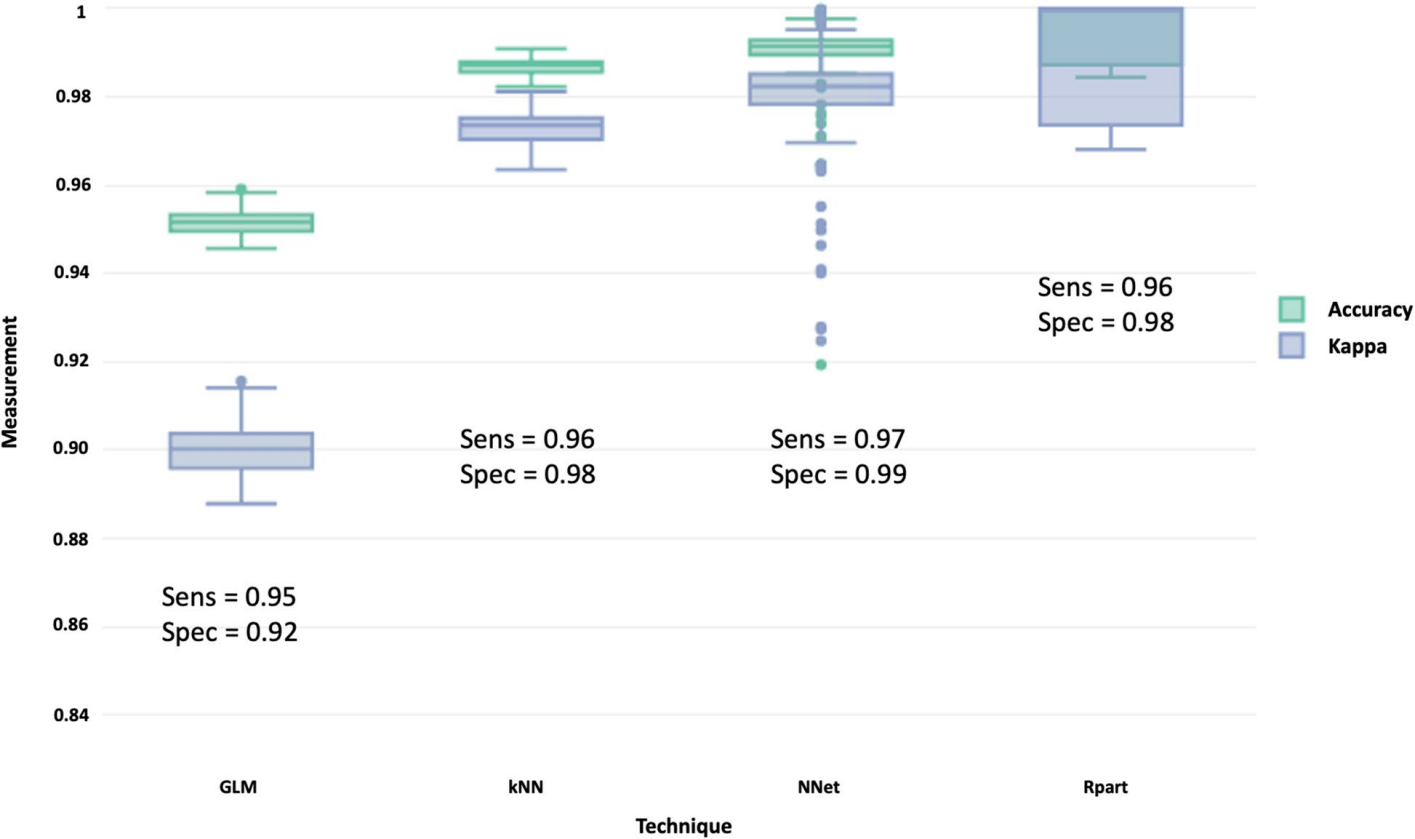
Box plot evaluates the performance metrics of different classifiers in the prediction of colorectal cancer based on dietary data

In supervised classifiers, all techniques performed very well where accuracy, kappa, sensitivity, and specificity were above 0.90 (Fig. 9). It appeared that the neural network performed better than the rest. By accounting the weight decay, the neural network model was optimal with a single layer of three hidden nodes, and we mapped out the schematic of the network, illustrated in Fig. 10. Sensitivity analysis also revealed seven features in the neural network model in future consideration (Fig. 11).

## Discussion

### Key findings

In this study, we show that colorectal cancer can be predicted based on a list of important dietary data using supervised and unsupervised machine learning approaches. The excellent level of prediction in the present study is congruent with previous findings where mis-classification only ranged from 1 to 2% [36, 37]. These machine learning models can be used both as an early tool to identify individuals at risk as well as predicting the clinical outcomes of colorectal cancer [38, 39].

Dietary control is one of the most effective protective and modifiable measures that the population can adopt for cancer prevention. Dietary features can signal clues of the likelihood of early-onset of specific type of colorectal cancer such as distal colon and rectum [40]. In fact, a systematic review of studies over a period of 17 years concluded that strong evidence linking dietary factors with CRC risk, however, specific food group components on this relationship, were limited [41]. The present study identified total fat, mono-unsaturated fats, linoleic acid, cholesterol, omega-6 as moderate to high correlated dietary features to positive colorectal cancer. In contrast, fiber and carbohydrates have negative correlation with colorectal cancer cases. These features reflects the evidence from precision nutrition that a combination of dietary parameters, particularly those in the healthy eating index (such as whole fruit, saturated fats, grains) are more accurate than single dietary index (such as glycemic index) is important in the modifiable behavior for cancer prevention [39, 42]. In addition, our text mining and apriori algorithm also indicated that vegetables, eggs, margarine, and cheese have great impacts on colorectal cancer.

Although all classifiers were very good predictors of CRC labels, artificial neural networks had the best accuracy and true positives and true negatives. The advantage of using neural networks over, for example, general linear models in cancer prediction, is having much lower uncertainty and better generalizability of the model [36, 43]. This is an important consideration since machine learning algorithms have increasingly been used in many medicine domains with varied success rates [44]. In addition, most or all data sets will have a clear imbalance between CRC and non-CRC labels. We used a smote technique to balance the data set, which otherwise, the machine learning models will predict all cases as non-CRC. Future work may need to consider controlling the sampling process to allow similar distribution of the two categories to minimize effects of down- or up- sampling. Another consideration is the age group of which this model is applicable. Unlike previous studies that account only for older people, this study includes younger adults in model training as well, therefore the models developed in this study may work well from young to older adults’ CRC prediction. With early and regular screening assisted by an optimal machine learning algorithm, the incidence of CRC can be reduced even further.

### Limitations

The strength of this study lies in the large datasets consisting of cases from seven major countries. Due to computational constraints, we randomly sampled observations to induce almost real-time estimates, model fits, and classification predictions. Some of the features that were not common had to be excluded from model development, which may result in confounding effects. The outcome label of CRC is based on detected cases and may not reflect early onset, new onset, or delayed onset of CRC as well as stratification of risk in different stages and types of CRC. Nevertheless, this study has narrowed down salient features that future researchers could consider in a more holistic approach, particularly, multi-dimensional that simultaneously accounts for diet, lifestyle, genetics, and related factors for CRC prediction.

## Conclusion

In this study, we concluded that a combination of unsupervised and supervised machine learning approaches can be used to explore the key dietary features for colorectal cancer prediction. To help with feasibility and practicality, the artificial neural network was found to be the optimal algorithm with misclassification of CRC of 1% and misclassification of non-CRC of 3%, for more effective cancer screening procedures. Furthermore, screening through dietary information can be used as a non-invasive procedure that can be applied in large populations. Using optimal algorithms coupled with high compliance to cancer screening will therefore significantly boost the success rate of cancer prevention.

## Supplementary Information

**Additional file 1.**

## Data Availability

The datasets generated and/or analyzed during the current study are available upon reasonable request from the lead author, Dr Hanif Abdul Rahman.
