# Supplementary material for: Machine learning-based colorectal cancer prediction using global dietary data
Source: BMC Cancer. 2023 Feb 10;23:144. doi: 10.1186/s12885-023-10587-x (PMC9921106; doi:10.1186/s12885-023-10587-x)
Supplement: Supplementary file 1 — Additional file 1. [file 12885_2023_10587_MOESM1_ESM.html]

supplementary.knit


**Machine Learning-based Colorectal Cancer Prediction using
Global Dietary Data**

Hanif Abdul Rahman

SOCR University of Michigan and Universiti Brunei Darussalam

hanifr@umich.edu;
hanif.rahman@ubd.edu.bn

Mohammad Ashraf Ottom

SOCR University of Michigan and Yarmouk University

maottom@umich.edu; ottom.ma@yu.edu.jo

Ivo D. Dinov

SOCR University of Michigan

statistics@umich.edu

# Supplementary 1

```
library(arules)
library(plotly)
library(dplyr)
library(arulesViz)

a.colrec <- read.transactions("df_trans_text2.csv", sep = ",", skip = 1, rm.duplicates=TRUE)

colrec_rule <- apriori(a.colrec, parameter=list(support=0.01, confidence=0.25, minlen=2))
```

```
## Apriori
## 
## Parameter specification:
##  confidence minval smax arem  aval originalSupport maxtime support minlen
##        0.25    0.1    1 none FALSE            TRUE       5    0.01      2
##  maxlen target  ext
##      10  rules TRUE
## 
## Algorithmic control:
##  filter tree heap memopt load sort verbose
##     0.1 TRUE TRUE  FALSE TRUE    2    TRUE
## 
## Absolute minimum support count: 629 
## 
## set item appearances ...[0 item(s)] done [0.00s].
## set transactions ...[3743 item(s), 62911 transaction(s)] done [0.06s].
## sorting and recoding items ... [15 item(s)] done [0.00s].
## creating transaction tree ... done [0.01s].
## checking subsets of size 1 2 3 done [0.00s].
## writing ... [19 rule(s)] done [0.00s].
## creating S4 object  ... done [0.00s].
```

```
sortedRule <- sort(colrec_rule)
x1   <- sortedRule@quality$support
y1   <- sortedRule@quality$confidence
z1   <- sortedRule@quality$lift
col1 <- sortedRule@quality$count
ruleNames <- paste0("Rule", c(1:length(sortedRule@quality$support)))

plot_ly(x = ~x1, y = ~y1, z = ~z1, color = ~z1, name=ruleNames) %>% 
  add_markers() %>% 
  layout(title=paste0("Arule Support-Confidence-Lift Plot (for all ", length(sortedRule@quality$support), " rules)"),
         scene = list(xaxis = list(title = 'Support'),
                     yaxis = list(title = 'Confidence'),
                     zaxis = list(title = 'Lift'))) %>% 
  hide_colorbar()
```

```
fi_rules<-subset(colrec_rule, items %in% "colrec_ca")
inspect(fi_rules)
```

```
##      lhs                                                                                                              rhs                                                             support confidence   coverage     lift count
## [1]  {Salad dressing, oil and vinegar, homemade}                                                                   => {colrec_ca}                                                  0.01071355  1.0000000 0.01071355  1.00000   674
## [2]  {Lettuce, salad with assorted vegetables including tomatoes and/or carrots, no dressing (Lettuce salad, NFS)} => {colrec_ca}                                                  0.01438540  1.0000000 0.01438540  1.00000   905
## [3]  {FAT BLEND FAT 75% FORTIFIED BREGOTT}                                                                         => {colrec_ca}                                                  0.02161784  1.0000000 0.02161784  1.00000  1360
## [4]  {Vegetable oil, olive}                                                                                        => {colrec_ca}                                                  0.01543450  1.0000000 0.01543450  1.00000   971
## [5]  {Egg, whole, fried, with fat (Scrambled egg, no milk added)}                                                  => {colrec_ca}                                                  0.01586368  1.0000000 0.01586368  1.00000   998
## [6]  {Vegetable oil,  canola and soybean}                                                                          => {colrec_ca}                                                  0.01789830  1.0000000 0.01789830  1.00000  1126
## [7]  {Beef, ground, medium, broiled}                                                                               => {colrec_ca}                                                  0.01827979  1.0000000 0.01827979  1.00000  1150
## [8]  {HARD CHEESE FETT 28%}                                                                                        => {colrec_ca}                                                  0.02867543  1.0000000 0.02867543  1.00000  1804
## [9]  {Butter, regular}                                                                                             => {colrec_ca}                                                  0.02824625  1.0000000 0.02824625  1.00000  1777
## [10] {Cheese, cheddar}                                                                                             => {colrec_ca}                                                  0.03024908  1.0000000 0.03024908  1.00000  1903
## [11] {Salad dressing, mayonnaise, commercial, regular}                                                             => {colrec_ca}                                                  0.02894565  1.0000000 0.02894565  1.00000  1821
## [12] {Egg, chicken, whole, fresh or frozen, raw}                                                                   => {colrec_ca}                                                  0.03619399  1.0000000 0.03619399  1.00000  2277
## [13] {Margarine, tub, composite}                                                                                   => {colrec_ca}                                                  0.04479344  1.0000000 0.04479344  1.00000  2818
## [14] {Shortening, household, unspecified vegetable oil}                                                            => {colrec_ca}                                                  0.06151547  1.0000000 0.06151547  1.00000  3870
## [15] {Egg, chicken, whole, fresh or frozen, raw,                                                                                                                                                                                  
##       Egg, whole, fried, with fat (Scrambled egg, no milk added)}                                                  => {colrec_ca}                                                  0.01193750  1.0000000 0.01193750  1.00000   751
## [16] {colrec_ca,                                                                                                                                                                                                                  
##       Egg, whole, fried, with fat (Scrambled egg, no milk added)}                                                  => {Egg, chicken, whole, fresh or frozen, raw}                  0.01193750  0.7525050 0.01586368 20.79088   751
## [17] {colrec_ca,                                                                                                                                                                                                                  
##       Egg, chicken, whole, fresh or frozen, raw}                                                                   => {Egg, whole, fried, with fat (Scrambled egg, no milk added)} 0.01193750  0.3298199 0.03619399 20.79088   751
```

```
plot(sort(fi_rules, by="lift"), method = "graph", engine = "htmlwidget",
     control=list(main = list(title="Grouped Matrix for the 14 Fentanyl-associated Rules")))
```

```
## Available control parameters (with default values):
## itemCol   =  #CBD2FC
## nodeCol   =  c("#EE0000", "#EE0303", "#EE0606", "#EE0909", "#EE0C0C", "#EE0F0F", "#EE1212", "#EE1515", "#EE1818", "#EE1B1B", "#EE1E1E", "#EE2222", "#EE2525", "#EE2828", "#EE2B2B", "#EE2E2E", "#EE3131", "#EE3434", "#EE3737", "#EE3A3A", "#EE3D3D", "#EE4040", "#EE4444", "#EE4747", "#EE4A4A", "#EE4D4D", "#EE5050", "#EE5353", "#EE5656", "#EE5959", "#EE5C5C", "#EE5F5F", "#EE6262", "#EE6666", "#EE6969", "#EE6C6C", "#EE6F6F", "#EE7272", "#EE7575", "#EE7878", "#EE7B7B", "#EE7E7E", "#EE8181", "#EE8484", "#EE8888", "#EE8B8B",  "#EE8E8E", "#EE9191", "#EE9494", "#EE9797", "#EE9999", "#EE9B9B", "#EE9D9D", "#EE9F9F", "#EEA0A0", "#EEA2A2", "#EEA4A4", "#EEA5A5", "#EEA7A7", "#EEA9A9", "#EEABAB", "#EEACAC", "#EEAEAE", "#EEB0B0", "#EEB1B1", "#EEB3B3", "#EEB5B5", "#EEB7B7", "#EEB8B8", "#EEBABA", "#EEBCBC", "#EEBDBD", "#EEBFBF", "#EEC1C1", "#EEC3C3", "#EEC4C4", "#EEC6C6", "#EEC8C8", "#EEC9C9", "#EECBCB", "#EECDCD", "#EECFCF", "#EED0D0", "#EED2D2", "#EED4D4", "#EED5D5", "#EED7D7", "#EED9D9", "#EEDBDB", "#EEDCDC", "#EEDEDE", "#EEE0E0",  "#EEE1E1", "#EEE3E3", "#EEE5E5", "#EEE7E7", "#EEE8E8", "#EEEAEA", "#EEECEC", "#EEEEEE")
## precision     =  3
## igraphLayout  =  layout_nicely
## interactive   =  TRUE
## engine    =  visNetwork
## max   =  100
## selection_menu    =  TRUE
## degree_highlight  =  1
## verbose   =  FALSE
```

```
# plot(fi_rules, method="graph", measure = "support", engine="htmlwidget", # nodeCol=rainbow(14),
#      shading = "lift", control = list(verbose = TRUE))
```

**Reference to Apriori protocol:**

DSPA module from SOCR
University of Michigan
